# Neurophysiological mechanisms of transcranial alternating current stimulation

**DOI:** 10.3389/fnins.2023.1091925

**Published:** 2023-04-05

**Authors:** Yuchen He, Shuang Liu, Long Chen, Yufeng Ke, Dong Ming

**Affiliations:** ^1^Academy of Medical Engineering and Translational Medicine, Tianjin University, Tianjin, China; ^2^Department of Biomedical Engineering, College of Precision Instruments and Optoelectronics Engineering, Tianjin University, Tianjin, China; ^3^Tianjin International Joint Research Center for Neural Engineering, Tianjin, China

**Keywords:** transcranial alternating current stimulation, neurophysiological mechanisms, neural entrainment, animal models, translation

## Abstract

Neuronal oscillations are the primary basis for precise temporal coordination of neuronal processing and are linked to different brain functions. Transcranial alternating current stimulation (tACS) has demonstrated promising potential in improving cognition by entraining neural oscillations. Despite positive findings in recent decades, the results obtained are sometimes rife with variance and replicability problems, and the findings translation to humans is quite challenging. A thorough understanding of the mechanisms underlying tACS is necessitated for accurate interpretation of experimental results. Animal models are useful for understanding tACS mechanisms, optimizing parameter administration, and improving rational design for broad horizons of tACS. Here, we review recent electrophysiological advances in tACS from animal models, as well as discuss some critical issues for results coordination and translation. We hope to provide an overview of neurophysiological mechanisms and recommendations for future consideration to improve its validity, specificity, and reproducibility.

## 1. Introduction

Neural oscillation is a prominent feature of neural activity’s temporal dynamics, correlated outcomes in both the health and clinical populations have shaped the core status of brain rhythms in neuroscience over the last decade (Engel et al., 2001; Schnitzler and Gross, 2005). Numerous studies have shown that cognition functions arise from the coordination of neural activity within intra-and inter-regional brain networks, which is dependent on the successful synchronization of various neural oscillations (Siegel et al., 2012).

In the field of brain science, brain stimulation by alternating current (AC) has a long history. AC brain stimulation at lower intensities was first used in 1950s by Anan’Ev and colleagues, which is known as “cranial electrotherapy stimulation” (Anan'Ev et al., 1957). It has also been used to treat tremor, dyskinesia and dyskinesia in this century (Limoge et al., 1999; Vitek, 2008). In 1986, Chan and Nicholson found that alternating electric stimulation can directly modulate brain activity (Chan and Nicholson, 1986). In recent years, transcranial alternating current stimulation (tACS) is gaining popularity as a non-invasive brain modulation for synchronizing electrophysiological rhythms, allowing for the establishment of causal links in the oscillation-cognition relationship (Uhlhaas and Singer, 2006). The conventional tACS stimulation pattern involves delivering weak sinusoidal currents at commonly constant frequencies through strategically placed electrodes, with appealing properties such as high operability, suitability for sham-controlled studies, and the absence of any serious adverse side effects (Ali et al., 2013; Alexander et al., 2019). Studies in animal models have been conducted to investigate the effect of tACS on neuronal polarization, which underpins the function of specific neurons and the cerebral cortex (Francis et al., 2003; Fujisawa et al., 2004; Deans et al., 2007). In humans, studies place electrodes above a targeted cortical region associated with specific functions, with the assumption that the underlying neuronal activity will be increased or decreased (Kar and Krekelberg, 2014; Riecke et al., 2015; Alekseichuk et al., 2016). Despite these findings, the mechanisms underlying the relatively high frequency-specificity remained unclear, which may limit our understanding of the temporal effect and its potential application for dynamic adaptivity enhancement (Nasr et al., 2022).

Animal models have been widely used to investigate the physiological mechanisms underlying tACS (Fröhlich and McCormick, 2010; Reato et al., 2010; Huang et al., 2021; Asamoah et al., 2022; Krause et al., 2022). In comparison to human interventions, these efforts using invasive approaches such as local field potentials (LFPs) and spiking activity of individual neurons have allowed for the direct evaluation of the effect of tACS in deep structure (Buzsáki et al., 2012). In this review, we summarize the current research on the tACS effect at the mesoscopic and macroscopic levels, as well as its possible neurophysiological mechanisms. We discuss some concerns about tACS efficacy and conclude with some recommendations with the aim of improving its robustness and replicability for future applications. We also propose potential avenues for translation to humans in order to advance our understanding of how tACS works.

## 2. Neurophysiological mechanism of tACS

### 2.1. Acute mechanism under subthreshold electric fields

In animal models, several studies have shown that the electric fields used by tACS are weak (~1 V/m), which is lower than the limited strength necessary to affect neuronal activity inside the brain (Vöröslakos et al., 2018; Asamoah et al., 2019). *In-vitro* electric field strengths as low as 0.2 V/m have been discovered to result in synchronous firing in phase with the applied stimulation (Reato et al., 2010); similar physiological effects have also been reported at an effective field value of 0.3 V/m (Deans et al., 2007). Taking in count the endogenous electric fields, Fröhlich and McCormick applied *in-vivo* electric fields at a threshold of 0.5 V/m and demonstrated that multi-unit activity (MUA) was synchronized to LFP oscillations *via* intracranial recordings from anesthetized ferrets (Fröhlich and McCormick, 2010). Subsequent *in-vitro* studies discovered that *in vivo*-like endogenous network activity influences the enhancing effect of tACS on endogenous oscillations (Schmidt et al., 2014).

To directly modulate the neuronal spike and local circuits within the target sites, a voltage gradient equal to or greater than 1 V/m is necessary, which is close to the upper limit as determined by invasive intracranial measurements in animal models (Ozen et al., 2010). The peak intensity of the applied field must be more than 4 V/m to exhibits multiunit neuronal firing (Up state) and exhibits multiunit neuronal (Down state) (Fröhlich and McCormick, 2010). In addition, a higher field intensity of 5–20 V/m is required for the cerebral blood flow alteration (Turner et al., 2021). Furthermore, the strength of the electric field necessary for specific physiological effects needs to be confirmed in both *in-vivo* and *in-vitro* studies, that can have distinct “activation” thresholds.

It is also known that a subthreshold electric field bi-directionally modulates its spontaneous spiking activity *via* resting membrane potential alterations (Deans et al., 2007; Radman et al., 2007). Besides, neurons also encode information in their temporal spiking patterns. Individual action potential temporal codes are reported to carry important information as well, and previous research has found that cortical neurons fire in synchrony with ongoing extracellular potential oscillations and task execution (Mehta et al., 2002; Harris et al., 2003). Understanding how such subthreshold electric fields affect spike timing and neural information processing in the central nervous system is therefore critical.

Several *in-vitro* investigations have been conducted to investigate the consequences of neuronal firing characteristics under subthreshold electric fields. For each 1 V/m of external field, a membrane potential change less than 0.5 mV depolarization of the cell bodies was found (Jefferys et al., 2003), and the degree of depolarization of the axon terminal is far more sensitive than that of the soma (Reato et al., 2010). Since such tiny membrane potential alternation is far below the spike initiation threshold, it is difficult to alter spiking activity in quiescent neurons under common *in-vitro* conditions. However, a 1 mV depolarization of a suprathreshold neuron can raise the firing rate by 6–9 Hz on average according to *in-vivo* investigations (Carandini and Ferster, 2000). The small but widespread depolarization contributes to a network-wide amplification of membrane potential perturbation and leads to the alternation in neuronal spike timing in the sustained networks, which are susceptible to electric fields. These depolarization amplification findings offered a potential network mechanism of tACS under subthreshold electric fields. The modulations of membrane potential and cortical excitability usually depend on the cortical excitation–inhibition balance, although the balance can be inverted by a short-duration, suprathreshold pulse-train (Khatoun et al., 2017). When these network effects are combined, the influences of a periodic AC field are not as simple as scaling frequency power in a given frequency range; they may be represented by sophisticated non-linear dynamics (Bestmann and Walsh, 2017).

In 2018, Liu et al. distinguished five mechanisms to explain the effects of tACS on neuronal and network activity: resonance, rhythmic resonance, temporal biasing of neuronal spikes, entrainment of network patterns, and imposed patterns (see Liu et al., 2018 for details). The authors stated that the physiological effect of tACS is determined by the interaction of endogenous and exogenous oscillations, and the strength of the required tACS field increases from stochastic resonance to the imposed patterns. These ideas support the existence of “response thresholds” for tACS. While these thresholds are possibly different for each specific case, this means that the tACS effectiveness is dependent on the combination of the brain region of interest and stimulation parameters including intensity and frequency. When the tACS frequency matches that of the exogenous field, the subthreshold effect of the exogenous extracellular field followed a frequency-specific resonance pattern and the endogenous oscillation can be successfully entrained (Asamoah et al., 2022). For the resonance pattern, very weak forces with resonant neuronal properties can modulate the spike timing of target neurons near the firing threshold during each cycle, causing cumulative effects over multiple cycles (Geisler and Goldberg, 1966; Francis et al., 2003; Deans et al., 2007).

Apart from the resonance pattern, tACS at non-preferred frequencies in intrinsic network necessitates stronger periodic stimulation for successful entrainment. For example, a 2 V/m frequency-matched tACS successfully entrained the intrinsic oscillation, and yet a 4 V/m field amplitude was required when frequency was not matched (Fröhlich and McCormick, 2010). An *in-vitro* study examined the correlation between native network activity and applied electric field (Schmidt et al., 2014). The authors found that the endogenous oscillations affect the role of exogenous fields and the main mechanism of tACS is possibly boosting the natural network rather than overriding, which questioned the supposed imposed mode. However, Krause recently discovered a competition mechanism in *in-vivo* studies (Krause et al., 2022). When tACS frequency is far from the endogenous dominant frequency, tACS and endogenous oscillations compete for spike timing control, with the entrainment effect determined by how ongoing oscillations influence neural activity. In specific, entrainment is reduced when neurons are strongly locked to ongoing oscillation, and it is reduced when neurons are strongly locked to ongoing oscillation. The origin spiking activity can be reversed and controlled at higher stimulation intensities.

Mc Laughlin et al. discovered a similar phenomenon, finding that when using 1 mA tACS, entrainment relative to baseline decreased, whereas when using 2 mA tACS, a comparable amount of synchronization to the stimulation waveform at a new phase was imposed (Mc Laughlin et al., 2022). According to the findings, increasing intensity causes neurons to desynchronize and re-train to the new phase. That is, the relative strengths of entrainment to the ongoing physiological oscillation and the tACS-induced electric field influence the entrainment effect. This can be thought of as an example of an imposed mode. In the imposed mode, the applied electric field has to be in opposition to the original endogenous electric field, and this mode necessitates a higher stimulation intensity.

### 2.2. Lasting mechanism of tACS

Aside from neuronal entrainment, the large-scale impact of tACS is linked to alterations in neuroplasticity (Korai et al., 2021). These alterations appear to be associated with tACS after-effects that remain longer than the stimulation duration (Vossen et al., 2015). However, the effects of tACS on neuroplasticity depend on certain experimental conditions. In a mouse research, 40 Hz-tACS, 20 min per day, had a substantial effect on the long-term enhancement of synaptic transmission in Alzheimer’s disease models after 2 weeks (Jeong et al., 2021). The study discovered that changes in protein synthesis, such as brain-derived neurotrophic factor (BDNF), are required for long-term plastic changes. There is an assumption that tACS causes neuroplasticity changes *via* long-term potentiation (LTP) and long-term depression (LTD; Zaehle et al., 2010). However, the direct induction of LTP and LTD in the context of tACS is still unclear. The potential effect of tACS in BDNF changes has been highlighted, as this neurotrophin can boost LTP by increasing synaptic responsiveness to high-frequency stimulation and physically by enhancing dendritic spine and arborization to facilitate synaptic transmission (Figurov et al., 1996; Amaral and Pozzo-Miller, 2007).

Human studies are also being conducted to investigate the role of BDNF-dependent plasticity in the after-effects of tACS. However, this effect now appears to be frequency dependent. According to one study, the Val66Met polymorphism, a single nucleotide polymorphism at codon 66 (Val66Met) in the BDNF gene, modulates the tACS effect in target oscillations under alpha tACS (Riddle et al., 2020). Furthermore, it was discovered that 20 Hz beta-tACS can induce NMDAR-mediated plasticity in the motor cortex and enhance cortical excitability as well as beta oscillations for at least 60 min (Wischnewski et al., 2019). Similarly, a human research discovered that after 20 min of tACS at the individual alpha frequency, the boosted alpha power can last for 70 min, compared to the sham-stimulation group (Kasten et al., 2016). However, the phenomenon was not detected using gamma tACS (Giustiniani et al., 2021). Fifty Hertz gamma-tACS was not successful in inducing an after-effect modulating sport performance in this study on healthy sports participants.

Furthermore, the intracranial electric field has been shown to affect glial cells and neurotransmitters in research on transcranial direct current stimulation (tDCS) (Monai et al., 2016; Gellner et al., 2021). To our knowledge, the effect of tACS on glial cells and neurotransmitters has yet to be investigated. It is still debatable whether after-effects are induced solely by neural plasticity or by a combination of neural plasticity and entrainment.

## 3. Factors influencing tACS efficacy

### 3.1. Detection methods

Detection methods play an important role in understanding tACS efficacy. For example, steady-state brain responses can be used to investigate the phase specificity of tACS. Previous research discovered that tACS had a long-lasting phase-specific enhancing or suppressing effect on steady-state brain responses (Fiene et al., 2020; Haslacher et al., 2022; Krause et al., 2022). When compared to spontaneous ongoing activity, tACS is expected to alter the phase of evoked brain activity with more difficulty as the steady-state signals always show dominant phase locking to rhythmic stimulation.

tACS neurophysiological studies typically employ inspection window lengths that correspond to the length of the entire stimulation period (Ozen et al., 2010; Asamoah et al., 2019; Krause et al., 2019). Mc Laughlin et al. discovered that neural entrainment detection is highly dependent on the observation window and epoch length (Mc Laughlin et al., 2022). Long epoch lengths, in particular, can detect entrainment while shorter windows cannot. When data collection time is limited, the researchers suggest that optimizing tACS paradigms to have fewer repetitions, but longer epoch durations will increase the likelihood of detecting an entrainment effect. Moreover, Haslacher et al. discovered a transient enhancement and suppression of oscillatory activity, as well as accomplishing millisecond-precise modulation of oscillations using a closed-loop approach, which provides an idea for reconciling the extensive variability of tACS (Haslacher et al., 2022). This predicts that standardization and refinement of spatio-temporal detection accuracy in detection methods will be beneficial for further investigation of the tACS effect.

### 3.2. Brain state

Much of the discussion regarding the validity of neural stimulation efficacy is linked to the state of endogenous oscillations (Bradley et al., 2022). tACS was shown to be capable of controlling transitions between different activity states (Kutchko and Fröhlich, 2013). A small periodic input, as stated in the resonance pattern, can cause neuron entrainment at the matched stimulation frequency (Riddle et al., 2022).

The role of endogenous oscillations in tACS effect is embodied in not only the degree to which neurons are entrained but also the phase difference between tACS and endogenous oscillations (Fiene et al., 2020; Haslacher et al., 2022; Krause et al., 2022). Evoked brain potentials have been used to study phase-dependent enhancement and suppression of endogenous oscillations (Fiene et al., 2020). These findings altogether suggested a dynamically adjusted protocol based on the current brain state. Recently, the closed-loop approach which allows for phase-locked to endogenous oscillations to selectively enhance or suppress ongoing activity, has been shown to improve modulation effects and robustness of tACS (Frohlich and Townsend, 2021; Haslacher et al., 2022; Nasr et al., 2022). By online adjustment of stimulation parameters, this brain-state dependent closed-loop protocol is expected to achieve dynamic adjustment and precise modulation.

However, the closed-loop protocol is challenging given the large artifacts caused by simultaneous signal acquisition and stimulation. There are efforts underway to carefully separate stimulation artifacts from physiological signals. Noury et al. proposed a mathematical model for the transfer function based on the amplitude and phase properties of stimulation artifacts (Noury et al., 2016; Noury and Siegel, 2017, 2018). Witkowski et al. used magnetoencephalography (MEG) in conjunction with synthetic aperture magnetometry (SAM) and successfully reconstruct responses during amplitude-modulated tACS (Witkowski et al., 2016). In addition, Ketz et al. found that pausing stimulation for a few seconds allowed for signal reconstruction in electroencephalogram (EEG) when trying to target low-frequency oscillations (Ketz et al., 2018). Later, Haslacher et al. used stimulation artifact source separation (SASS) to separate EEG signals from artifacts (Haslacher et al., 2021, 2022). While strategies for rejecting artifacts in other situations are still being investigated.

**Figure 1:**
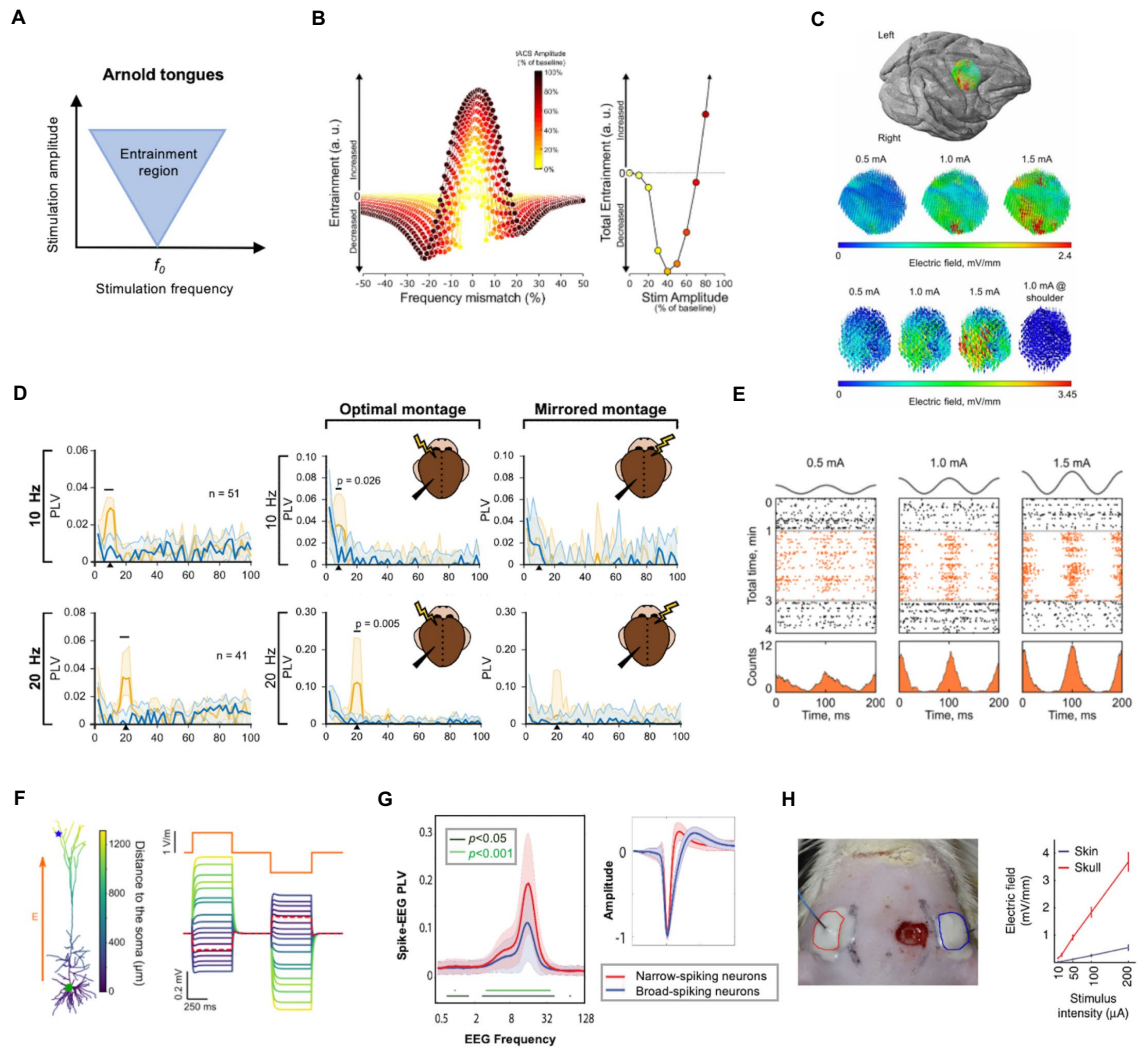
Neurophysiological mechanisms and essential factors for tACS. **(A)** Illustration of Arnold tongue. The inverted triangle shapes the possible stimulation amplitude and frequency parameter combinations. **(B)** The entrainment changes at different combinations of frequency mismatch and stimulation intensity (left) and total entrainment are calculated by integrating the left curves within 2 Hz bins (right). It is illustrated that enhanced oscillation occurs when the stimulation frequency closely matches the endogenous oscillation; however, even a minor mismatch can result in decreased oscillation when the stimulation amplitude is relatively weak. **(C)** Electrical field intensity distribution in one monkey on the target brain area. The field distributions had the same orientation and relative spatial relationships as the intensity increased linearly from 0.5 to 1.5 mA. **(D)** Phase-locking value (PLV) spectra of target neurons during tACS at 10 and 20 Hz during sham (blue line) and active tACS duration (orange line). The tACS effects of target neurons are specific to the stimulation frequency (left column) and location (right column). Only around the stimulation frequency did neural entrainment occur at each frequency (horizontal black lines indicate significant bands). Furthermore, when compared to the contralateral side, tACS on the ipsilateral side of signal recording showed significant entrainment. **(E)** tACS-induced entrainment of one representative neuron from an awake monkey at 0.5, 1, and 1.5 mA intensities. The spike rate (lower row) and time course (upper row: black dots for pre−/post-stimulation duration, orange dots for stimulation duration) revealed an increase in neuron spikes and clustering to the peak of the sine wave as the intensity increased during the stimulation period, indicating a dose-dependent manner. **(F)** Electric field sensitivity in a passive pyramidal cell model (the blue star represents the apical dendrites, and the green circle represents the soma). The orange line represents the stimulation current. The figure depicts how the induced field differed from neuron morphology. **(G)** The synchrony between network-scale oscillations and interneurons varies according to neuron type. Right: Two types of neurons were identified based on spike characteristics: narrow-spiking (red line) neurons and broad-spiking neurons (blue line). Narrow-spiking neurons had higher PLV than broad-spiking neurons. **(H)** A diagram of transcutaneous stimulation in rodent research (left) and a comparison of the electric field values of transcutaneous and subcutaneous stimulations at the same stimulus intensities (right). The field value is heavily influenced by the stimulation pattern. Subfigures B, C, D, E, F, G and H have been adapted with the authors’ and publisher’ permission.

The neural entrainment from tACS can be shaped as “Arnold tongues” (Frohlich and Riddle, 2021). As shown in Figure 1A, the inverted triangle shapes the possible entrainment areas under specific stimulation intensities and frequencies and explains the dynamics between endogenous oscillation and tACS field (Ali et al., 2013; Frohlich and Riddle, 2021). The entrainment area is centered on the intrinsic frequency of the stimulated network and radiates to the surrounding bands. Moreover, as the stimulus intensity increases, so does the range of entrainment and with a broader range. Recently, the Arnold tongues were observed in an *in-vivo* study on awake ferrets (Huang et al., 2021). In this study, triangular tongues were demonstrated by the synchronization map between single-units and tACS, implying that particular parameter combinations of tACS give a reasonable approach for mode design.

Most *in-vivo* experiments are conducted in anesthetized animals. Anesthesia, on the other hand, can alter neural dynamics and brain metabolism (Paasonen et al., 2018). Given the change in network structures with awake states, care should be taken when translating or comparing these findings to human studies (Krause et al., 2022). As shown in Figure 1B, the endogenous oscillation can be increased when tACS is precisely matched to the dominant frequency, whereas it can be reduced even with minor frequency detuning. Entrainment was found to be increased when the stimulation intensity exceeded about 66% of the amplitude of the ongoing oscillation. In awake states, endogenous networks may reflect more complicated oscillations, inadvertently strengthening or weakening the effects of externally applied tACS fields (Laufs, 2008; Johnson et al., 2020).

Generally, a detailed state assessment may be required during the stimulation process. During a rodent study, Khatoun et al. checked the anesthesia level by checking the toe-pinch reflex and promptly provided intraperitoneal drug perfusion to ensure a relatively stable oscillation structure during the stimulation process (Khatoun et al., 2017). It is possible that the same stimulation protocols will produce different electrophysiological responses depending on the current state of the brain. Thus, the reproducible results caused by uncontrolled state-dependency phenomenon increase the difficulty of obtaining reliable stimulation outcomes. This can be explained by the aforementioned response thresholds and the entrainment area in varied Arnold tongues under changing endogenous structures.

### 3.3. Factors influencing entrainment

The specificity of the tACS effect is an important premise for therapeutic applications, as it can provide relative target modulation within neuronal circuits (Kanai et al., 2008; Ali et al., 2013). The field primarily affected the spike of neurons beneath montages based on the expected settings (Figure 1C). Apart from spatial specificity, a frequency-specific pattern was found. As shown in the right column of Figure 1D, entrainment of neurons beneath target region only significantly increased at or around the tACS frequency (Krause et al., 2019). Furthermore, the study revealed spike timing entrainment in a dose-dependent manner (Johnson et al., 2020; Figure 1E). This finding was consistent with the network perspective discussed previously. In addition to the single entrainment within the region of interest, a unique cross-frequency phase-amplitude coupling (PAC) stimulation pattern has emerged (Helfrich et al., 2016; Jones et al., 2020; Grover et al., 2021). tACS can manipulate inter-regional phase synchronization and yield cross-frequency coupling between endogenous and exogenous activity in this manner, providing a unique application for tailored stimulation. Grover et al. recently reviewed the likely mechanisms underlying the tACS effect on PAC and associated therapeutic applications (Grover et al., 2021); they will not be covered in detail here due to space and scope constraints.

One study revealed that cortical excitation varied non-linearly with increasing intensity of 140 Hz tACS (Moliadze et al., 2012). In addition, subsequent research discovered a lower effectiveness in generating membrane polarization at higher stimulation frequency (Deans et al., 2007; Khatoun et al., 2017). These findings appear to point to a mechanism of mutual cancelation of inhibitory and excitatory effects. Considering the high firing frequency determined by repolarization and lower membrane time constant in inhibitory neurons, they may be more sensitive to 140 Hz tACS at lower intensities than excitatory neurons. The effect of tACS on cortical excitation necessitates systematic titration of stimulation parameters, and non-linear modulation deserves careful consideration, given the change in the time required for a neuron to cross the threshold for action potential generation caused by tACS (Radman et al., 2007). According to the current state of cortical excitation-inhibition balance, a weak external electric field is more likely to affect neurons close to the threshold and synchronize their spiking time. Together, these results suggest that frequency-matched oscillatory electrical fields mostly affect the temporal structure of the neural activity without major changes in the overall activity level.

However, increased power after tACS in the target region is widely regarded as evidence of successful neuronal entrainment (Vinck et al., 2010; Hill et al., 2011). The direction of the applied AC field influences the modulation pattern of neuronal firing, and the entrainment effect is also related to the direction of the electric field. Previous research has confirmed the definitive role of entrained neurons’ biophysical properties, such as morphology, phase preference, and orientation (Aspart et al., 2018; Toloza et al., 2018; Tran et al., 2022; Figure 1F).

Neuron type is another factor that may influence tACS responsiveness. An alpha-tACS study in awake head-fixed ferrets revealed that synchrony between field oscillations and single-unit spikes was stronger in narrow-spiking neurons than in broad-spiking neurons, possibly due to stronger endogenous coupling between fast-spiking inhibitory interneurons and alpha oscillation (Huang et al., 2021; Figure 1G). These findings lay the foundation for prominent entrainment of target neurons as well as network-scale oscillations during transcranial stimulation. A tACS study on morphologically realistic neurons also suggested that the applied electrical field may primarily target large pyramidal neurons (Tran et al., 2022). Overall, precise neural entrainment is a significant challenge that limits the replicability of tACS.

## 4. Concerns about tACS effectiveness

The assumption that tACS of the human brain works similarly to animal experiments is risky. Transcutaneous delivery is the most common application of tACS in humans, whereas animal models are always subcutaneous. Under transcutaneous pattern, only a small portion of the applied current enters the deep brain structure. According to Vöröslakos et al., the electric field on the scalp is significantly stronger than that in the cortex. They demonstrated in this study that nearly 75% of the scalp-applied current was attenuated in the tortuous gyrus of the brain in human cadavers (Vöröslakos et al., 2018). The authors also conducted *in-vivo* experiments on rats, delivering subcutaneous and transcutaneous electric stimulation *via* similarly sized electrodes as in the human samples, and they reported an 80 ± 5% current loss under the transcutaneous condition regardless of current intensity (Figure 1H).

Likewise, Ozen et al. delivered electrical fields to rodents *via* electrodes on the surface of the skull while simultaneously recording intracranial neural activity (Ozen et al., 2010). They observed an increase in the percentage of phase-locked neurons to external stimulation as the intensity increased. The electric fields inside the brain are large enough to modulate brain activity even if the majority of current is lost due to shunting. However, the differences in effective stimulation intensity with various tACS delivery approaches should be evaluated further. Given the massive current shunting, the subcutaneous approach should provide smaller stimulation intensity than the transcutaneous approach.

However, concerns have been raised regarding potential confounders (Raco et al., 2014). The physiological response could be caused by peripheral nerve stimulation or other peripherally mediated effects such as retinal stimulation, and due to the high conductivity of the eyes and a relatively low-resistance pathway, both close and distance montages could induce a current to the retina (Laakso and Hirata, 2013). Indeed, even with a small fraction of the total current, the visual information distribution and processing during stimulation are sufficient to generate subjective sensations. Such sensations are known as phosphenes, and they are a common side effect of stimulation at 10–20 Hz (Kanai et al., 2008; Kar and Krekelberg, 2012). Individual stimulation intensity below the phosphene-threshold is one available method in human studies to avoid that phenomenon (Pogosyan et al., 2009; Feurra et al., 2011). Considering the amount of visual processing influences phosphene perception, stimulating in a brighter environment or with eyes open and administering a visual task during stimulation may be beneficial for weakening phosphene perception (Ahn et al., 2019; Alexander et al., 2019; Frohlich and Riddle, 2021). It is also reported that amplitude-modulated tACS (AM-tACS) showed no phosphene with stimulation intensities of up to 2 mA (Thiele et al., 2021).

Thus, neurons may be entrained by upstream areas rather than by the current directly present in the area of interest. Asamoah et al. recently distinguished the transcranial and transcutaneous mechanisms of tACS in rodents and human volunteers (Asamoah et al., 2019). They conducted four separate experiments by selectively blocking the pathways and found that the tACS directly affect the peripheral nerves while having an indirect effect on motor cortex activity. Combined with the transcranial-only results, which showed that the weak electric field generated by tACS at around 1 V/m can cause significant entrainment in cortical neurons, there are still significant challenges in explaining tACS effects in nonmotor systems.

In response to the above complications, recent studies on non-human primates have provided some support for the efficacy of tACS. Krause et al. created two montages of recording and stimulation sites that produced equivalent stimulation through the retinal area by reflecting an optimal and mirrored pattern (Krause et al., 2019). They found that neural entrainment occurred only at the optimized pattern, not the mirrored one. Because the mirrored montage should produce a similar sensation, the absence of spiking activity modulation in that case rules out the possibility that the effects of neuronal activity using the optimal montage are indirect. Similarly, Johnson et al. conducted a control block by peripherally mediated effects in awake primates (Johnson et al., 2020). When tACS electrodes were attached to the right upper arm, there was no entrainment, and this finding favored the direct effect of tACS on the brain. In line with these findings, Vieira et al. devised a novel experiment in which somatosensory input was blocked by applying a topical anesthetic to the skin surrounding each stimulation site (Vieira et al., 2020).

## 5. Translation

### 5.1. Translation across species

Although field values below 1 V/m are considered effective as above mentioned, however, voltage gradients described in animal research cannot be directly compared to human investigations. Disparities in brain volume, anatomy, and skull thickness can all have a significant impact on physiological effects, posing significant challenges to translation. In fact, the field strengths in animal models are several times larger than values reported in human studies. In non-human primates, Krause et al. found that when two macaque monkeys were given 2 mA tACS (4 mA peak-to-peak amplitude) through personalized electrode montage during an arousal and motivational state, the peak electric field strength in the hippocampus and basal ganglia was 0.28 V/m in one monkey and 0.35 V/m in the other (Krause et al., 2019). Moreover, Johnson et al. reported comparable field strength in awake primates (Johnson et al., 2020). It was discovered to be slightly stronger through invasive measurement, reaching as high as 1.33 V/m during 1.5 mA tACS. These studies took great care in measuring electric fields and ensuring that the amplitudes used were comparable to human studies.

In humans, *in-vivo* intracranial attempts are being made to determine the spatial and temporal distribution of intracranial electric fields induced by tACS, which provide a valuable picture of how the applied alternating current flows in the brain. In 2016, Opitz et al. used stereotactic EEG to measure the spatial distribution of applied electric fields (Opitz et al., 2016). Maximum field strengths in human brains can reach 0.36 V/m in one participant and 0.16 V/m in the other for 1 mA stimulation currents. In 2017, Huang et al. expanded the sample size to 10 humans and provided extensive field estimates of the entire brain in conjunction with calibrated modeling (Huang et al., 2017). They found that the maximal electric field values are around 0.4 V/m and 0.16 V/m in more extended regions under 2 mA scalp current, which is the generally reported maximum stimulation strength in human research. Moreover, Louviot et al. recently demonstrated similar field distributions while investigating the electrical field in deep brain structures using high-density tACS (HD-tACS) (Louviot et al., 2022).

**Figure 2:**
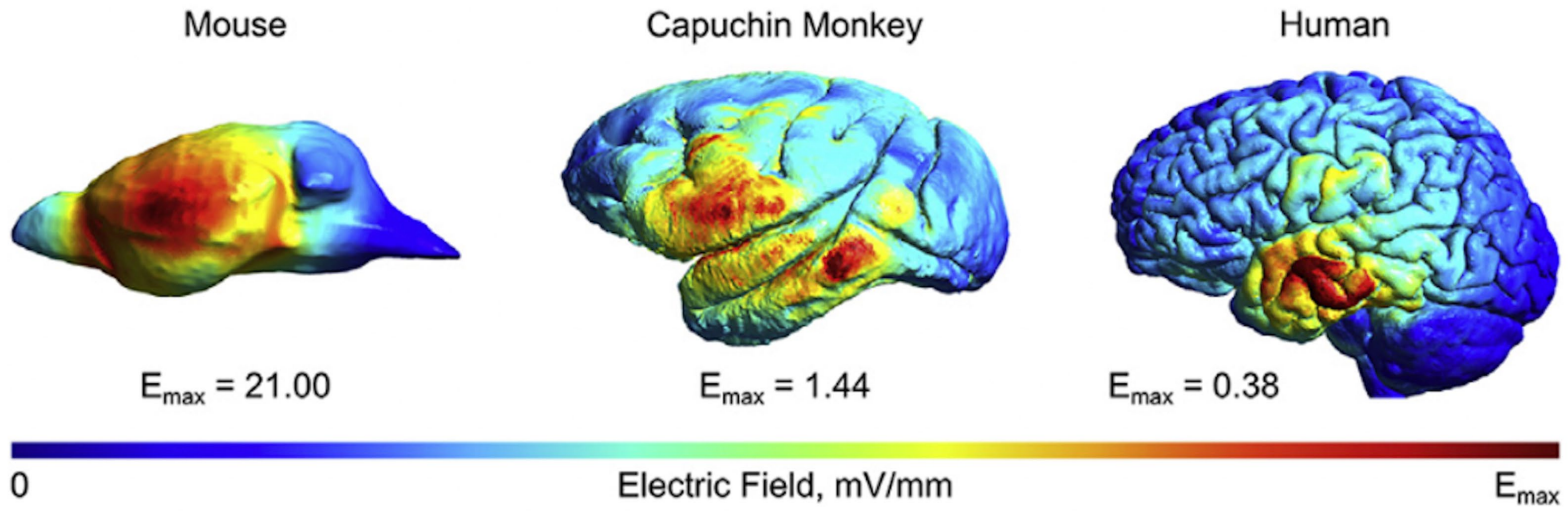
Different field values across species. A comparison of the normalized electrical fields induced by transcranial electrical stimulation in mice, monkeys, and humans. The electrode montage was identical across species, and the current intensity was set to the same level. It can be seen that the maximum field strength of the three models decreases exponentially with increasing brain volume. The figure has been adapted with the authors’ and publisher’ permission.

In a comparative study, computational modeling with finite element models (FEM) was applied across studies in animals and humans, and it was discovered that field strength was inversely proportional to head size (Alekseichuk et al., 2019). Compared to rodents, field values in non-human primates with larger head sizes were relatively comparable to humans under matched stimulation conditions, with a value difference of around 1 V/m (Figure 2). This finding allows for a quantifiable scaling metric to enable intuitive comparisons between human and animal models in translational studies.

### 5.2. Translation across techniques

Another barrier to translation is at the technical level, where results obtained through several methodologies might be difficult to translate directly. Some tACS *in-vitro* studies demonstrated electric field magnitudes of up to 20 V/m, which is 10–20 times greater than the electric field observed in *in-vivo* studies (~1 V/m) (Chan and Nicholson, 1986; Deans et al., 2007). Parallels between these two approaches should be drawn with caution. Computational models are anticipated to offer a thorough understanding of how the applied current diffuses from the stimulation site to the entire brain in this context and to bridge the gap between different levels of observation (Bai et al., 2013; Laakso and Hirata, 2013). The current diffusion process assists us in understanding the mechanism of action of tACS and provides an answer to the question of how tACS could alter brain activity effectively and reproducibly.

The increasingly advanced computational models certainly assist in understanding which regions are most stimulated by certain stimulation patterns, the proportion of stimulation diffusion, and which regions are unaffected by a particular electrode montage. The ideal electrode design is then selected based on the predicted field distribution (Dmochowski et al., 2011; Ruffini et al., 2014). While, more emphasis is suggested to place on the distribution of the electric field under different stimulation conditions, for example, the relative value of the field strength. To achieve a viable tACS application, it has been suggested that the electrode placement accuracy be less than 1 cm (Opitz et al., 2018).

Besides, modeling studies rely heavily on the construction of three-dimensional head models and the determination of conductivity (Saturnino et al., 2019; Louviot et al., 2022). To acquire the electrical field distribution, the head modeling requires *in-vivo* validation. Huang et al. found that a full-head clinical magnetic resonance imaging (MRI) scan, from neck to crown, is required to get reliable findings (Huang et al., 2017). Furthermore, while calibrating using *in-vivo* intracranial recordings, the authors discovered that variances in skull layers or conductivity variations induced by current direction in white matter had no effect on accurate model prediction. However, there are still doubts regarding whether the computational models based on these conductivities can truly give reliable information for improving electrode location and electric field distribution, which must be confirmed in *in-vivo* investigations (Kasinadhuni et al., 2017).

## 6. Directions for future studies

**Figure 3:**
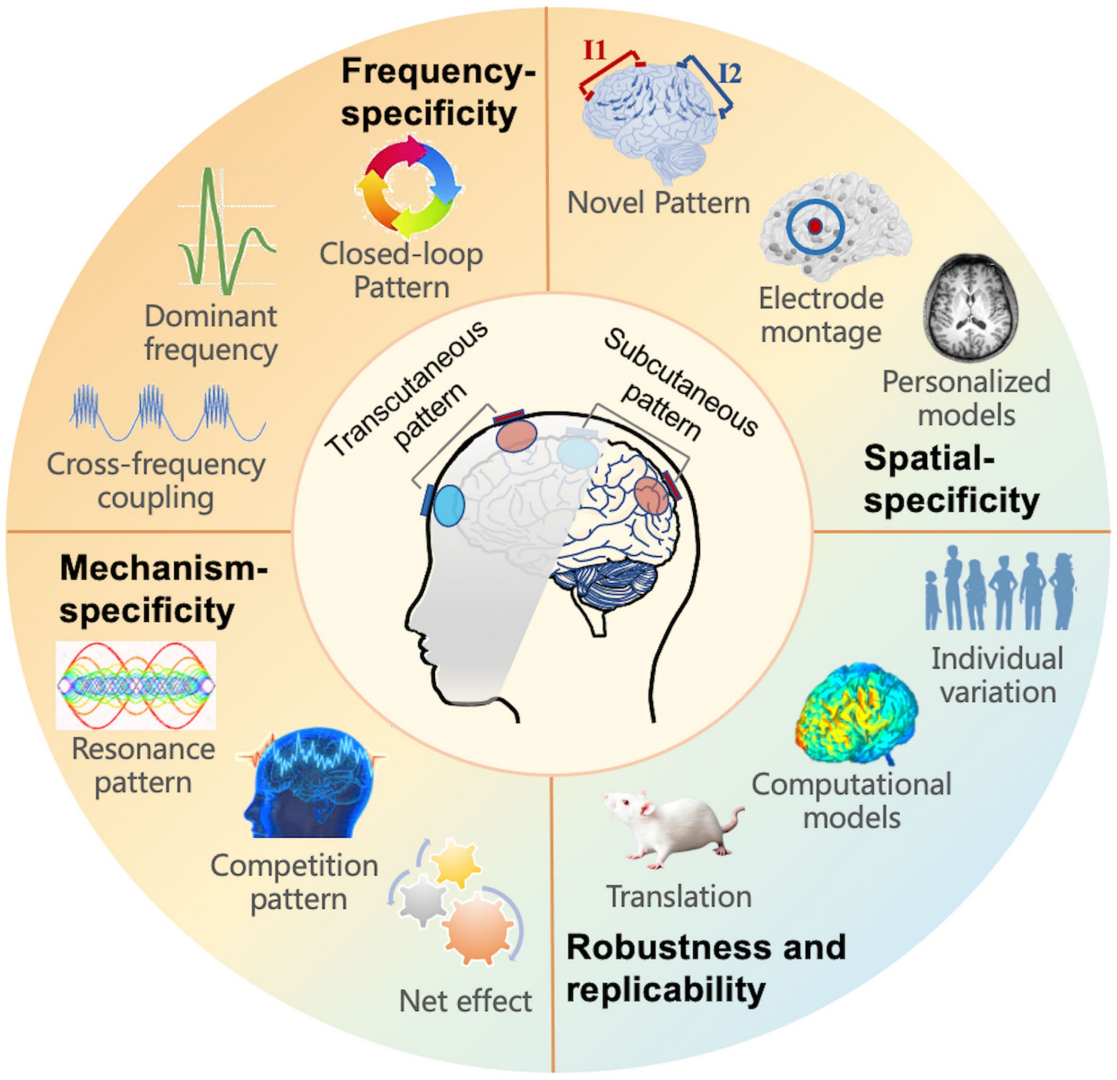
Future directions for tACS, including four aspects to take into account: frequency, spatial, mechanism-specificity, as well as robustness and replicability. The subfigure introduces the implicated or prospective study directions.

For future applications of tACS, a comprehensive knowledge of tACS, including electrophysiological effectiveness and rational experimental design, is required (Figure 3). We summarized various prospective perspectives in the domains of innovative and individual stimulation patterns, with the goal of concluding with suggestions for optimal modulation.

### 6.1. Novel stimulation protocol

The conventional saline-soaked sponge electrodes of square centimeter scale in human research may lead to more current being shunted, and increases the likelihood of inducing skin sensation (Turi et al., 2014). From this perspective, a focused and small montage is likely to weaken peripherally mediated effects by limiting the shunted current (Khatoun and Mc Lauglin, 2017). Numerous unique tACS stimulation patterns have appeared during the past 10 years, including high definition tACS with muti-montage around the central single electrode and the ring montage made up of a tiny center electrode and an encircling ring electrode (Preisig et al., 2021). These new paradigms encourage field focality, and allow for adequate management of the spatial peak fields around the target area (Saturnino et al., 2017). There is also great effort being put into developing tACS protocols that stimulate target brain regions, such as high-density transcranial alternating current stimulation (HD-tACS) (Helfrich et al., 2014) and temporal interference stimulation (Grossman et al., 2017), to improve the spatial specificity of tACS.

The customizable current waveform is essential for temporal intention given the large parameter space for tACS. For example, random noise, AM-tACS and non-sinusoidal current emerge for target neural rhythm (Terney et al., 2008; Fröhlich and McCormick, 2010; Negahbani et al., 2018). To comprehend the electrophysiological response foundation of novel stimulation modalities, as well as non-neuronal possibilities such as neurotransmitter metabolism, trophic factors, and immune system components, further experiments in animal models are required (Liu et al., 2018).

### 6.2. Personalized stimulation strategy

Before undertaking human investigations, it is very desirable to expand clinical applications of effective medicines in pre-clinical animal models. To optimize electrode positions for a desired field distribution, computational models could therefore make use of data from animal and human studies (Dmochowski et al., 2011; Sánchez-León et al., 2018). MRI models that are specifically tailored to the particular patient may be useful. Wang et al. observed that with the same tACS intensity, a personalized simulation pattern accurately predicted the electrical field (Wang et al., 2021).

Individual variation in neuroanatomy (scalp, muscle, and skull thickness and scalp-to-cortex distance), cortical excitability, and specific inhibitory and excitatory circuits of local networks, each of which may change susceptibility to an external electric field, is a major factor altering the modulation effect between individuals (McConnell et al., 2001; Krause and Cohen Kadosh, 2014; Kasten et al., 2019). According to studies, the induced electric field and inherent oscillation properties can account for between 54 and 65% of the variability in the tACS effect (Zanto et al., 2021). According to our research, human participants with lower endogenous activity can benefit more from particular tACS, whereas subjects with beginning performance following stimulation showed mild or even negative alterations (Liu et al., 2022).

The peak frequency of the intended endogenous oscillation might be used as the stimulation frequency for each participant as an additional strategy to reduce individual variations because the dominant frequency of endogenous oscillations differs between individuals (Chiang et al., 2011; Ruhnau et al., 2016). Likewise, it is important to take into account the stimulus goal as a consideration. A recent research on a patient with depression used customized intracranial brain stimulation to target a particular circuit and discovered dependable mood improvements (Scangos et al., 2021). This method enables the replication of findings between species and laboratories. Additionally, the psychological condition of patients should be taken into consideration while evaluating the value of tACS, since the way in which stimulation is perceived subjectively can significantly affect how it works. Although this is reasonably simple to detect in people, it is more difficult to notice in animal models, which presents difficulties for efforts at cross-species and cross-laboratories translation.

## 7. Conclusion

In recent decades, research has demonstrated the important role of neural oscillations in information exchange and transmission between brain networks. Over the last 10 years, tACS has emerged as an indispensable neuromodulation tool for understanding the link between behavior and brain oscillations. tACS has demonstrated a unique role in clinical intervention and improvement of cognitive function. However, the electrophysiological mechanisms of tACS are still unclear, and further exploration and understanding of micro-mechanisms are necessary from animal models. It is worth mentioning that the spatial and temporal targeting is a fundamental stage in the application for the treatment of psychiatric illness. Preclinical experiments are also necessary to enable parameter titrations and customized stimulation techniques. While inconsistent stimulation settings and tactics may be the main cause of inconsistent translation outcomes between laboratories and species, improving this pipeline will be essential for improving the possibility of translating research from animal models to people.

We carefully examined the neurophysiological mechanisms behind tACS in this review. The application of animal models opens up new avenues for human study by enabling the validation and back-translation of human findings, which, in theory, will result in innovative treatment methods. Future research should concentrate on understanding the very complicated mechanisms behind common brain illnesses as well as non-invasive treatment approaches. It is particularly important to take into account the security and protection of study animals, necessitating that experimenters balance animal welfare and create animal models with a high level of translational validity (Homberg et al., 2021).

## Author contributions

All authors listed have made a substantial, direct, and intellectual contribution to the work and approved it for publication.

## Funding

This work was funded by National Natural Science Foundation of China (No. 81925020 and 19JCYBJC29200).

## Conflict of interest

The authors declare that the research was conducted in the absence of any commercial or financial relationships that could be construed as a potential conflict of interest.

## Publisher’s note

All claims expressed in this article are solely those of the authors and do not necessarily represent those of their affiliated organizations, or those of the publisher, the editors and the reviewers. Any product that may be evaluated in this article, or claim that may be made by its manufacturer, is not guaranteed or endorsed by the publisher.
